# Supplementary material for: Extracellular Nanovesicles Secreted by Human Osteosarcoma Cells Promote Angiogenesis
Source: Cancers (Basel). 2019 Jun 5;11(6):779. doi: 10.3390/cancers11060779 (PMC6627280; doi:10.3390/cancers11060779)
Supplement: Supplementary file 1 [file cancers-11-00779-s001.pdf]

Supplementary Materials

Extracellular Nanovesicles Secreted by Human Osteosarcoma Cells Promote Angiogenesis

Francesca Perut, Laura Roncuzzi, Nicoletta Zini, Annamaria Massa and Nicola Baldini

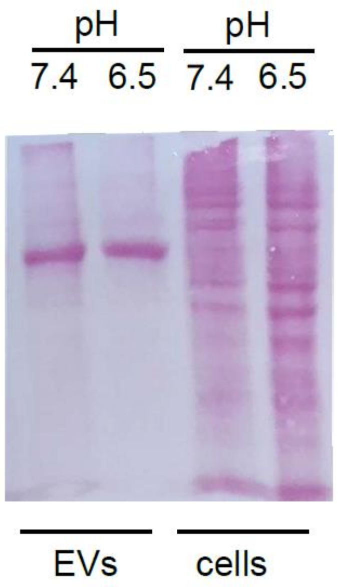

Figure S1. Ponceau S stained nitrocellulose membrane.

Supplementary Materials for Whole blot related to Figure 1 (panel b).

Osteosarcoma (OS)-derived extracellular nanovesicles (Evs) characterization.

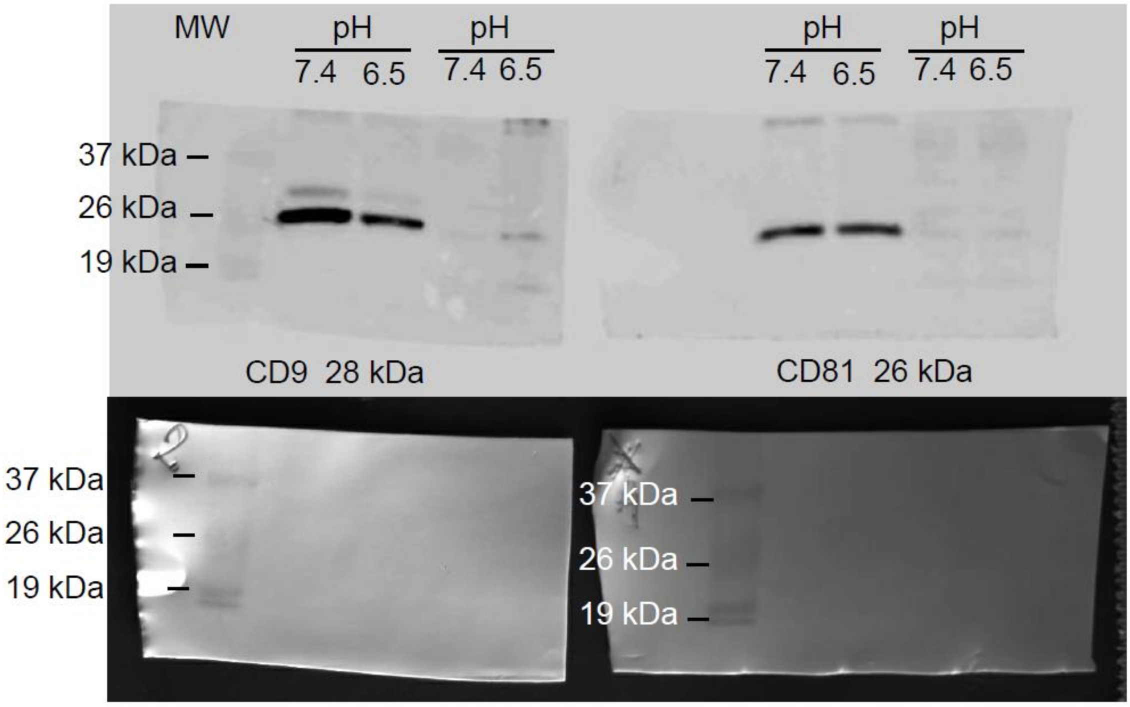

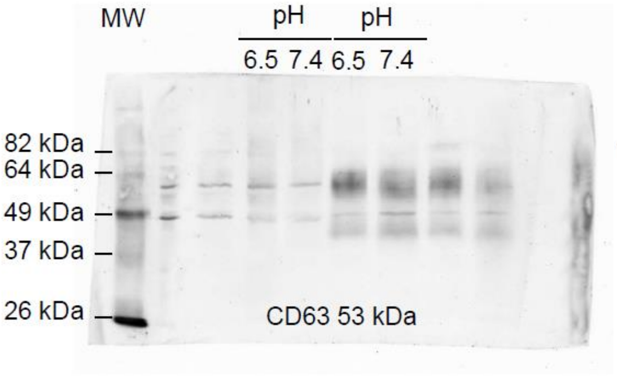

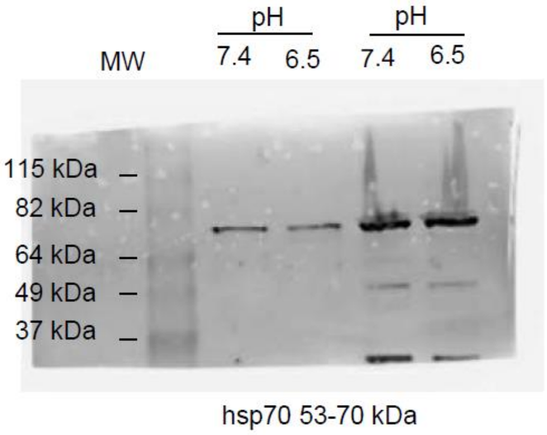

© 2019 by the authors. Licensee MDPI, Basel, Switzerland. This article is an open access article distributed under the terms and conditions of the Creative Commons Attribution (CC BY) license (http://creativecommons.org/licenses/by/4.0/).
